# Lack of Delta-Sarcoglycan (*Sgcd*) Results in Retinal Degeneration

**DOI:** 10.3390/ijms20215480

**Published:** 2019-11-04

**Authors:** Andric C. Perez-Ortiz, Martha J. Peralta-Ildefonso, Esmeralda Lira-Romero, Ernesto Moya-Albor, Jorge Brieva, Israel Ramirez-Sanchez, Carmen Clapp, Alexandra Luna-Angulo, Alvaro Rendon, Elva Adan-Castro, Gabriela Ramírez-Hernández, Nundehui Díaz-Lezama, Ramón M. Coral-Vázquez, Francisco J. Estrada-Mena

**Affiliations:** 1 Massachusetts General Hospital, Division of Surgery, 55 Fruit St, Boston, MA 02214, USA; andric@aya.yale.edu; 2 Laboratory of Epidemiology and Public Health, Yale University School of Public Health, 60 College St, New Haven, CT 06510, USA; 3 Facultad de Química, Universidad Nacional Autónoma de México, 04510 Ciudad de México, Mexico; martha.janneth2830@gmail.com; 4 Laboratorio de Biología Molecular, Universidad Panamericana, Escuela de Medicina, Donatello 59 Insurgentes Mixcoac Benito Juárez, 03920 Ciudad de México, Mexico; elira@up.edu.mx; 5 Facultad de Ingeniería, Universidad Panamericana, Augusto Rodin 498, 03920 Ciudad de México, Mexico; emoya@up.edu.mx (E.M.-A.); jbrieva@up.edu.mx (J.B.); 6 Sección de Estudios de Posgrado e Investigación, Escuela Superior de Medicina, Instituto Politécnico Nacional, 11340 Ciudad de México, Mexico; iramirez@up.edu.mx; 7 Instituto de Neurobiología, Campus UNAM-Juriquilla, Universidad Nacional Autónoma de México (UNAM), 76230 Querétaro, Mexico; clapp@unam.mx (C.C.); elva.adan@gmail.com (E.A.-C.); gaby.farmafesc@gmail.com (G.R.-H.); 8 Departamento de Neurociencias, Instituto Nacional de rehabilitación, México-Xochimilco, No.289. Arenal de Guadalupe, 14389 Ciudad de México, Mexico; lunangulo@gmail.com; 9 Institut De La Vision, Sorbonne Universites, F-75012 Paris, France; alvaro.rendon@inserm.fr; 10 Department of Physiological Genomics, Ludwig-Maximilians-Universität München, Großhaderner Str. 9, 82152 Planegg-Martinsried, Germany; nundehuidiaz@gmail.com; 11 Subdirección de Enseñanza e Investigación, Centro Médico Nacional “20 de Noviembre”, Instituto de Seguridad y Servicios Sociales de los Trabajadores del Estado, 03100 Ciudad de México, Mexico

**Keywords:** dystrophin-associated protein complex, delta-sarcoglycan, knock-out mice, age-related macular degeneration, geographic atrophy, retinal degeneration

## Abstract

Age-related macular degeneration (AMD) is the leading cause of central vision loss and severe blindness among the elderly population. Recently, we reported on the association of the *SGCD* gene (encoding for δ-sarcoglycan) polymorphisms with AMD. However, the functional consequence of Sgcd alterations in retinal degeneration is not known. Herein, we characterized changes in the retina of the Sgcd knocked-out mouse (KO, *Sgcd*^−/−^). At baseline, we analyzed the retina structure of three-month-old wild-type (WT, *Sgcd^+/+^*) and *Sgcd*^−/−^ mice by hematoxylin and eosin (H&E) staining, assessed the Sgcd–protein complex (α-, β-, γ-, and ε-sarcoglycan, and sarcospan) by immunofluorescence (IF) and Western blot (WB), and performed electroretinography. Compared to the WT, *Sgcd^−/−^* mice are five times more likely to have retinal ruptures. Additionally, all the retinal layers are significantly thinner, more so in the inner plexiform layer (IPL). In addition, the number of nuclei in the KO versus the WT is ever so slightly increased. WT mice express Sgcd-protein partners in specific retinal layers, and as expected, KO mice have decreased or no protein expression, with a significant increase in the α subunit. At three months of age, there were no significant differences in the scotopic electroretinographic responses, regarding both a- and b-waves. According to our data, *Sgcd^−/−^* has a phenotype that is compatible with retinal degeneration.

## 1. Introduction

Age-related macular degeneration (AMD) is a leading cause of irreversible blindness worldwide. Despite being a prevalent cause of vision impairment, there are regional disparities in prevalence and incidence, suggesting that genetics and environmental factors might influence the lifetime risk of AMD [1]. There are two major genes and gene families, *HTRA1, ARMS2* and *CFH*, which are consistently associated with the development of the disease [2,3,4]. However, these associations differ across countries and ethnicities, arguing for additional genomic loci whose contribution has not been elucidated so far [4,5,6,7]. In previous work, we showed that polymorphisms in a gene from the dystrophin-associated protein complex (DAPc), δ-sarcoglycan (*SGCD*), significantly increased the odds of AMD, and was especially associated with the geographic atrophy phenotype of the disease among Mexican patients [8].

The DAPc was firstly characterized in muscular tissues, whose impairment leads to a myriad of muscular dystrophy phenotypes [9]. Among patients affected with either Duchenne’s (dystrophin deficiency), Becker’s (milder phenotype), or limb-girdle muscular dystrophies (LGMD2, Sgcd deficiency), there are reports of altered retinal function, as measured by electroretinography (ERG), as well as scarce reports on visual impairments [10,11]. In Duchenne cases, for instance, ~47% have red–green color vision defects, depending on the locus of the deletion [11]. Furthermore, there are also changes in the full-field ERG, especially significant decreases in mesopic positive peaks for both ON and OFF stimuli and photopic peaks for ON stimulus [10]. Part of these effects might be due to a so-far unknown critical role of the DAPc protein complex in maintaining retinal structure and function [10,11].

We and others previously characterized the expression of the components of the DAPc in the murine retina and its independence from the dystrophin [12]. We specifically described β-, γ-, δ-, ε-sarcoglycans (Sg), and sarcospan (Sspn) expression along the outer and the inner limiting membranes, probably in the Müller cells, and in the ganglion cells axons [12]. We also have noted the importance of these proteins in retinal homeostasis in Müller glial cells, since δ-, γ-Sg, β-dystroglycan, α-1-syntrophin, and dystrophin 71 (Dp71) aid in the formation of the retinal blood barrier, and its absence leads to retinal edema [13].

Building upon these findings, we aimed to characterize the changes in the retinal structure, function, and protein expression of the DAPc in the *Sgcd^−/−^*mouse. We chose this model since, to date, there are no reports fully characterizing the retinal alterations of cases with limb-girdle muscular dystrophy (LGMD2) regarding retinal function. Moreover, we have evidence that *Sgcd* is a pivotal gene for the development of AMD. Hence, the consequences of its absence, especially in the ERG and the DAPc expression (indirect maintenance of the retinal blood barrier), are critical for researching and tailoring future therapeutic approaches.

## 2. Results

### 2.1. Changes in Retinal Structure among Three-Month-Old Mice Knocked-Out (KO) and Wild-Type (WT)

**Figure 1:**
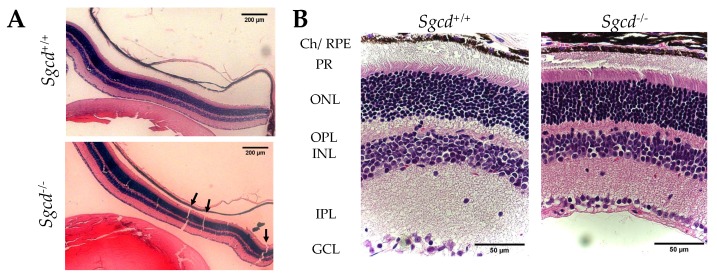
Retinal morphology of three-month-old *Sgcd^−/−^* and *Sgcd^+/+^* mice. (**A**) Representative retinal cross-sections stained with hematoxylin and eosin (H&E) at 5× magnification. Black arrows point to regions of retinal tearing. (**B**) Representative retinal cross-sections stained with H&E at 20× magnification. Ch/RPE: Choroids, retinal pigmented epithelium; PR: photoreceptor layer; ONL: outer nuclear layer; OPL: outer plexiform layer; INL: inner nuclear layer; IPL: inner plexiform layer; GCL: ganglion cell layer.

At first glance, in retinal slides stained with hematoxylin and eosin (H&E), the knocked-out (KO, *Sgcd^−/−^*) mouse has a thinned and frail retina compared to the wild type (WT, *Sgcd^+/+^*). Upon microscopic observation, there were abundant solutions of continuity in the retina of the knocked-out mouse (KO) compared to the WT (Figure 1A, arrows). These observations were not associated with the slide-processing technique, since these results were replicated multiple times by experienced technicians. Instead, these results are in support of a frailer tissue. On higher magnification, there is a marked decrease in retinal thickness in the *Sgcd^−/−^* group (Panel B). To further dissect these observations, experienced technicians quantified each layer. Moreover, we corroborated these measures with an automated method of image processing in Mathlab (Mathworks, Natick, MA, USA) and built an algorithm to quantify any solution of continuity (See Methods, Section 4.3). The structural characterization of both groups is further described in Table 1.

Overall, there is a significant reduction in retinal thickness. On average, KO mice have ~100 µm less than wild type (*p* < 0.0001). This difference holds on different magnitudes for all retinal layers except for the ganglion cell layer (GCL) (Table 1). The lack of statistical significance in GCL is likely related to its monocellular layer structure or the difficulty in measuring it precisely with an operator or an automated method. Moreover, our results suggest that the retina of the KO mice is frail since they had a median number of retinal ruptures of 13, while the WT mice had no quantifiable solution of continuity in the H&E slides (Figure 1 Panel A, Table 1).

Moreover, to examine the thinned retina of the KO mice, we estimated the outer- and inner-nuclear cell layer densities (per 100 µm^2^) using our automated method. Based on the number of data collected, there are significant differences in cell density in both layers between groups. The mean differences by genotype are displayed in Table 1.

### 2.2. Sarcoglycan Complex Expression and Localization by Immunofluorescence among Three-Month-Old Mice

**Figure 2:**
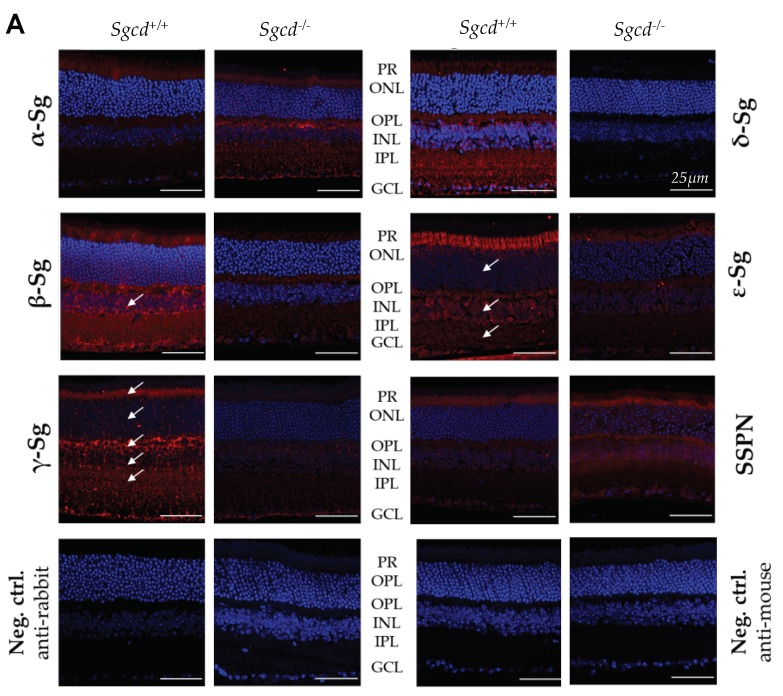

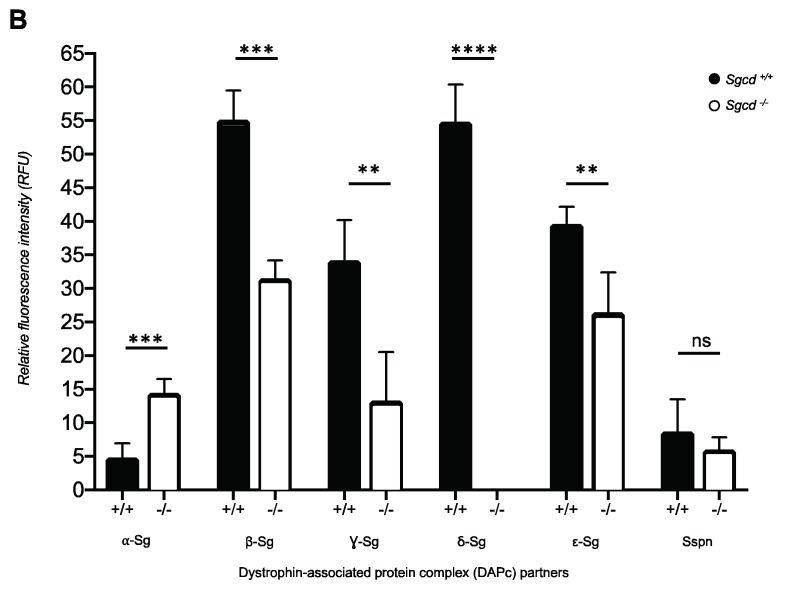
Indirect immunofluorescence against the sarcoglycan–sarcospan protein complex in retinal sections of three-month-old *Sgcd^−/−^* (*n* = 3) and *Sgcd^+/+^* (*n* = 3) mice. (**A**) Representative images were taken at 20 × 3.63 magnification by confocal microscopy. Sg: sarcoglycan; Sspn: sarcospan; Neg. ctrl: negative control; PR: photoreceptor layer; ONL: outer nuclear layer; OPL: outer plexiform layer; INL: inner nuclear layer; IPL: inner plexiform layer; GCL: ganglion cell layer; ns: not significant at the 0.05 level. Scale bar: 50 µm. (**B**) Immunofluorescence intensities quantifications, means ± standard error. Overall significance from an ANOVA and pairwise comparison by Student’s *t*-test with Sidak–Bonferroni corrected α values.

To characterize the impact of Sgcd protein’s absence in the KO mice, we began by examining the distribution and expression of the sarcoglycan-sarcospan (Sg-Sspn) protein complex composed by, α-, β-, γ-, δ-, ε-, and ζ-Sg and Sspn [14,15]. Here, we only studied the first five, since ζ-Sg was the last to be discovered, and we had limited access to antibodies. Excluding α-Sg, the rest of the sarcoglycans were significantly under-expressed in the KO compared to the WT, as demonstrated by immunofluorescence assays (Figure 2A). We observed the distribution of the Sg in the WT murine retina localized to the photoreceptor (PR), outer and inner plexiform, and ganglion cell layers in retinal slides (Figure 2 arrows). Our positive findings in the PR layer could indicate expression in the PR cells itself or, most likely, their positivity in the outer limiting membrane (Müller glial cells). So far, we are limited to indicate the approximate region of Sg-Sspn expression in the murine retina. Regardless, the absence of *Sgcd* leads to a significant decrease in the Sg-Sspn complex. Unlike the other Sgs, α-Sg is overtly expressed in the KO compared to the WT. This subunit is localized to the outer plexiform and possibly GCL (Figure 2). Regarding Sspn, the KO seems to have a downwards expression, but our measurements were not significantly different from the WT.

### 2.3. Changes in Sarcoglycan Complex Protein Expression in Dissected Retinas of Three-Month-Old Mice

**Figure 3:**
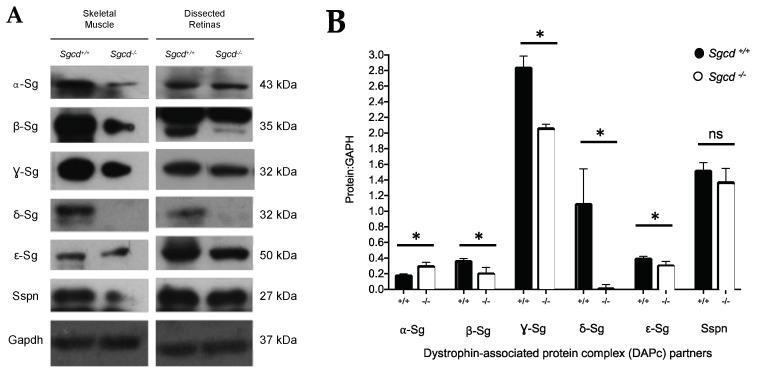
Expression of the Sg-Sspn complex of retinal and skeletal muscle in three-month-old *Sgcd*^−/−^ (*n* = 12) and *Sgcd*^+/+^ (*n* = 12) mice. (**A**) Western blotting for sarcoglycan–sarcospan protein complex in murine-dissected retinas and skeletal muscle (gastrocnemius). (**B**) Glyceraldehyde 3-phosphate dehydrogenase (Gapdh) normalized protein expression of the complex subunit by genotype. ns: not significant at the 0.05 level; *: *p*-value > 0.05.

To further support our findings of the Sg-Sspn complex expression in retinal slides by immunofluorescence (IF), we extracted retinas from WT and KO mice and performed Western blot to analyze the protein levels of the complex. We used skeletal muscle lysates from both WT and KO mice as a positive control for the experiment. The Sgs and Sspn are expressed in the WT mouse retina. In support of our immunofluorescence results, the *Sgcd* protein knock-out leads to a down-regulation of all subunits in the protein complex except for α-Sg (Figure 3A right panel and Figure 3B). Similarly, Sspn was not significantly different among genotypes. The uncut scans of our Western blot images are presented in the Supplementary Figures S1–S7.

The α-Sg/Gapdh median normalized expression is significantly higher in the *Sgcd^−/−^* mice compared to the control (~0.14 units of difference, *p* = 0.0286, Table 2). The rest of the Sgs show a lower level in the *Sgcd^−/−^* mice, with the most evident change observed in γ-Sg (−0.7787, *p* = 0.0286). A detailed description of the normalized protein expressions of the Sg-Sspn complex is tabulated in Table 2. Sspn was not significantly different between genotype groups.

### 2.4. Effect of Sgcd Knock-Out in Retinal Function by Electroretinography among Three-Month-Old Mice

**Figure 4:**
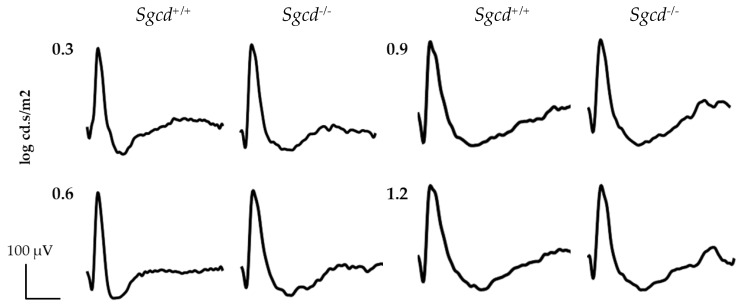
Electroretinographic scotopic responses in three-month-old *Sgcd^−/−^* and *Sgcd^+/+^* mice at different light intensities after dark adaptation.

Finally, to assess the significance of *Sgcd* loss on retinal function, we performed scotopic electroretinography (ERG) assessments in both groups at different light intensities (Figure 4). We evaluated both genotypes at three months of age. At this age, no significant differences were evident in retinal function in a-, b-, and c-waves (Figure 4).

## 3. Discussion

Age-related macular degeneration is the leading cause of irreversible blindness in developed countries. AMD prevalence is rising due to population aging and a lack of effective treatment [1,16]. We recently described a possible association between the *SGCD* gene (rs931798 polymorphism) with increased odds of geographic atrophy AMD and a haplotype configuration with lowered odds of disease (GATT: rs970476, rs931798, rs140617, rs140616) [8]. Thus, to investigate the contribution of this gene to the pathogenesis of AMD, here we analyzed a KO mouse and based on our findings proposed it as a model of retinal degeneration. Our results showed a statistically significant reduction in the retinal thickness of *Sgcd^−/−^* compared to the *Sgcd*^+/+^. Interestingly, this retinal thinning is similar to the clinical findings of geographic AMD (GA/AMD) and diabetic retinopathy cases [17,18,19]. We speculate that δ-Sg′s absence leads to a loss of structural proteins or the dysregulation of signaling pathways that could lead to a thinner retina. This assumption is based on our earlier finding of *SGCD*′s polymorphisms associated with GA/AMD. [8] A new bioinformatic report described Sgcd′s involvement in tissue regeneration, developmental growth, cell proliferation, and differentiation [20]. All these functions could explain our finding of a thinner frailer retina. However, further studies are warranted. Likewise, there was a significant increase in retinal frailty and outer and inner nuclear layer nuclear densities. Regarding the protein expression of the Sg-Sspn complex, we observed a significant downwards expression of β-, γ-, and ε-Sg in the KO. These findings in these subunits are comparable to the skeletal and cardiac muscle expression in the *Sgcd^−/−^* mouse [21,22]. We are first to report these protein alterations in the retina for this mouse.

The Sg-Sspn complex expression is partially independent of dystrophin in the mouse retina [12]. Based on its relationship and cellular membrane location, this complex might provide retinal stability through the binding of the cytoskeleton with the extracellular matrix [12,23]. Consequently, a decrease in the expression of the complex’s subunits could be associated with higher retinal frailty, as observed here. Interestingly, we did not detect a significant change in Sspn retinal expression. This finding is different from the effect of knocking out *Sgcd* in skeletal and cardiac muscle, where it leads to a decrease or absence of Sspn [24]. *Sspn* gene deletion does not modify the expression of the Sg complex, which implies that its expression and assemblage are independent, and our results are in support of this hypothesis [24]. We observed by Western blot and immunofluorescence an increased α-Sg expression in the retina of the *Sgcd^−/−^* mouse. This is the first report of α-Sg over-expression in the murine retina. Our results suggest that this protein could play a role in the morphological findings when *Sgcd* is absent. α-Sg might compensate for a decrease in ε-Sg expression, since α- and ε-Sg share an amino acid identity of almost 50% [22]. Hence, our findings foster the hypothesis that these proteins might constitute different complexes in the mouse retina. However, future studies should test this assumption.

Finally, we did not find evidence of any difference in retinal function, as measured by ERG, between the *Sgcd* KO and the WT. We possibly under-detected any functional difference owing to the early age of our mice. Commonly AMD (characterized by retinal degeneration) is diagnosed around age 65, and its prevalence increases with age. However, clinically, in early AMD phenotypes, there is an anatomical thinning of retinal layers [17,18,25]. It remains unclear if, in earlier stages of the disease, there is a measurable impact in retinal function. Another limitation of our study is the broad assessment of the Sg-Sspn protein distribution in retinal slides. So far, our positive results in the photoreceptor layer could indicate that some proteins of the complex could be either expressed in this cell or Müller glial cells. However, we feel that our approach with two complementary methods (IF and WB) is a first step in the complete assessment of the role of the Sg-Sspn in the murine retina. Future studies could comprehensively study the molecular underpinnings surrounding retinal degeneration in the *Sgcd*^−/−^ mouse.

## 4. Materials and Methods

### 4.1. Study Design and Ethical Approval

We performed an animal study of 42 C57BL/6J mice, half wild-type (WT, *Sgcd^+/+^, n* = 21) and half knock-out (KO, *Sgcd^–/–^*). All mice were either male or female of 8–10 weeks bred in-house at the Animal Care of Universidad Panamericana. The *Sgcd*^−/−^ mice used in the protocol were provided by Dr. Ramón M. Coral-Vazquez. Every animal was fed with regular chow and kept under standard light–dark cycles. All the experimental procedures were approved by the Internal Committee for the Care and Use of Laboratory Animals (Approval number: CICUAL-04/15-08-2014, dated March 2017). Furthermore, we observed both The Association for Research in Vision and Ophthalmology (ARVO) Statement for the Use of Animals in Ophthalmic and Vision Research [26] and the Code of Practice for the Housing and Care of Animals Bred, Supplied or Used in Scientific Purposes [27].

### 4.2. Mice Genotyping

#### 4.2.1. DNA Extraction

To genotype our mice, we began by extracting genomic DNA by tailing. Approximately 3 mm of the tip of the tail was cut under local anesthesia and collected in 1.5-mL tubes. Then, we immediately washed three times all the tissues with phosphate-buffered saline (PBS) 1X and dehydrated with 90 µL of 5 mM of NaCl solution. Shortly after, the tail was crushed with an aluminum shank. Then, we performed cellular digestion by adding 37.5 µL of 10% Sodium Dodecyl Sulfate (SDS) and 307.5 µL of saturated NaCl. Tubes were mixed using a vortex and centrifuged for 10 min at 4 °C and 14,000 rpm. The supernatant was recovered in new 1.5-mL collection tubes, and DNA was precipitated with 1 mL of 4 °C absolute ethanol. Afterward, we centrifuged the DNA for 1 min at full speed and removed the supernatant. We washed the pellet with 70% ethanol and centrifuged for 10 min at full speed. The supernatant was decanted for allowing DNA to dry for 5 min. Finally, genomic DNA was resuspended in 20 µL of DNAse free water. DNA concentrations and purity were quantified using a Multiskan™ GO Spectrophotometer (Thermo Fisher Scientific Inc., Wilmington, DE, USA). After quantification, the DNA was diluted to a concentration of 150 ng/µL. Finally, to evaluate DNA integrity, we prepared a 1% agarose (Thermo Fisher Scientific Inc., Wilmington, DE, USA) gel stained with 0.5 µg/mL ethidium bromide.

#### 4.2.2. PCR Reactions

We prepared a PCR mix (10 µL) using a final concentration of 0.2 mM dNTPs (Thermo Fisher Scientific Inc., Wilmington, DE, USA), 0.08× of each primer, 0.5 U/µL DreamTaq DNA polymerase, and 1× DreamTaq Buffer (Thermo Fisher Scientific Inc., Wilmington, DE, USA). One microliter (~150 ng of DNA) was added to the PCR mix. The amplification conditions were: 30 s at 95 °C (melting), 30 s at 67 °C (annealing), and 60 s at 72 °C (extension) for 35 cycles. Amplicons were run on a 2% agarose gel stained with 0.5 µg/mL ethidium bromide, and were compared to the GeneRuler 100 base pairs (bp) DNA ladder (Thermo Fisher Scientific Inc., Wilmington, DE, USA). Per primer design [5′ GCTTTCCCTGCTCCTGGTTCATTT 3′ (sense), 5′ TTCCCACTTCTTGACCCTGTCGTT 3′ (antisense) and 5′ ACCTTGCTCCTGCCGAGAAAGTAT 3′ (neomycin cassette)], our amplicons of 700 bp corresponded to an *Sgcd*^+/+^ mouse and 1014 bp for the neomycin cassette corresponded to an *Sgcd^−/−^* mouse.

### 4.3. Morphological Analysis

To characterize the effect of Sgcd on retinal structure, we performed a morphological analysis by firstly measuring the retinal thickness (4.3.1), then evaluating for retinal frailty (4.3.2), and finally estimating the nuclei density in the outer (ONL) and inner nuclear layers (INL) (4.3.3). We began by enucleating our groups following the ARVO statement and peer-reviewed protocols [26,28]. Both eyes from each mouse were fixed in 4% paraformaldehyde overnight at 4 °C. Then, all tissues were dehydrated and embedded in paraffin. We performed serial transversal sections of 2.5 µm, taking the optic nerve as a reference. Half of the slides were stained with hematoxylin and eosin (H&E) for aim 4.3 and the rest for immunofluorescence (4.4).

#### 4.3.1. Retinal Thickness

We quantified the full retinal thickness and each layer by light microscopy at ×10 magnification using the AxioVison Rel. 4.8 software (Carl Zeiss Inc., Thornwood, NY, USA). On average, we obtained three images and three measurements of each unit of analysis (eye). Then, we measured, per standard protocol, 500 µm to the right and left of the optic nerve.

#### 4.3.2. Frailty Index

We previously observed that *Sgcd^−/−^* mice tissues, especially neural, were frail compared to wild type [29]. We here aimed to quantify if there were any differences in the light microscopy of full-retinal segments. We began by assessing H&E stained slides, which was done by experienced blinded technicians, taken at ×5 in MatLab (Mathworks, Natick, MA, USA). We visualized each image using a script of Matlab (Mathworks, Natick, MA, USA), where we selected and extracted all retinal layers. Then, we transformed these photographs from the RGB color model to the HSV color model. Then, we obtained a grayscale image, representing the luminance of the RGB image, allowing us to highlight the regions of interest [30,31]. Considering the shape and orientation of the retina in the eye, we applied image-processing techniques to estimate a curve that encodes the shape and direction of the analyzed layer of the retina. Following this curve, we can analyze the changes in the intensity of the pixels along the given direction, where the discontinuities will be those pixels where its intensity difference with its neighbors is greater than a threshold. Finally, we quantified the number of discontinuities to describe the grade of the frailty of this layer.

#### 4.3.3. Nuclei Density

To quantify ONL and INL nuclear density, we analyzed H&E slides taken at 40× magnification in MATLAB (Mathworks, Natick, MA, USA). We began by visualizing each image firstly on the script. Then, we transformed each photograph from RGB to HSV format to obtain a grayscale image similar to 4.3.2. Afterward, we selected and extracted from the original image the area between the ONL and INL. These images were binarized to cluster pixels into two categories, (1) layer nuclei equal to zero and (2) cytoplasm and extracellular matrix equal to 255, in the grayscale. Then, we applied a segmentation algorithm to extract the outer plexiform layer to obtain two images corresponding to the nuclear layers. Finally, we quantified, using the method of Wang et al. [32], in each layer all the pixels belonging to the nuclei. We examined four images per eye taken around 500 µm of the right and left of the optic nerve.

### 4.4. Immunofluorescence of the Sarcoglycan-Sarcospan Complex

Briefly, eyes embedded in paraffin were sectioned (2.5 µm) and mounted on positively charged slides (Thomas Scientific Inc., Swedesboro, NJ, USA). Sections were deparaffinized in 100% xylene and hydrated with 100%, 96%, 90%, and 70% ethanol dilutions and distilled water for 3 min each. Then, the slides were transferred to a plastic Coplin jar containing citrate buffer (0.1 M, pH 6), heated to 100 °C, and then transferred to a steamer for 25 min for antigen retrieval. Later, the Coplin jar was cooled to room temperature for 30 min. Slides were placed into a dark wet chamber, and the eye areas were delimited using a hydrophobic pen (Thermo Fisher Scientific Inc., Wilmington, DE, USA). Sections were washed with PBS 1× three times and permeabilized with 1% Triton X-100 for 30 min and blocked with universal blocking solution (BioGenex Inc., Fremont, CA, USA) for 1 h. Then, sections were washed with PBS 1× three times and incubated with anti-alpha (GeneTex Inc., Irvine, CA, USA; Cat. #GTX87192; 1:100), -beta (GeneTex Inc., Irvine, CA, USA; Cat. #GTX55795; 1:100), -gamma (GeneTex Inc., Irvine, CA, USA; Cat. #GTX117176; 1:100), -delta (GeneTex Inc., Irvine, CA, USA; Cat. #GTX32871; 1:100), and -epsilon (GeneTex Inc., Irvine, CA, USA; Cat. #GTX33494; 1:100) sarcoglycan antibodies and anti-SSPN antibody (Abcam, Cambridge, UK; Cat. #ab186730; 1:100) at 4 °C overnight. On the next day, the sections were washed three times with PBS 1× and were incubated for 90 min at room temperature with goat anti-rabbit Cy3 (Thermo Fisher Scientific Inc., Wilmington, DE, USA; Cat. #A10520; 1:500) or goat anti-mouse Cy3 (Thermo Fisher Scientific Inc., Wilmington, DE, USA; Cat. #A10521; 1:300). Finally, slides were washed with PBS 1× and covered with mounting medium containing 4′,6′-diamino-2-phenylindole (DAPI) for nuclei staining (Abcam, Cambridge, UK; Cat. #ab228549). Images from at least six samples per group were taken using a Nikon Confocal A1R+ STORM Microscope (Nikon Inc., Minato, Tokyo, Japan). The images were analyzed by measuring the fluorescence intensity with ImageJ software (ImageJ2, National Institutes of Health, Bethesda, MD, USA).

### 4.5. Western Blot of the Sarcoglycan-Sarcospan Complex

Mice retinas were dissected and placed in 100 µL of lysis buffer (20 mM of Tris-HCl pH 7.4, 140 mM of NaCl, 2 mM of Ethylenediaminetetraacetic Acid (EDTA), 5 mM Na3VO4, 30 mM of NaF, 1% Tritón X-100, 0.1% SDS) supplemented with 10 µL of protease (Roche, Basel, Switzerland; Cat. #04693124001) and phosphatase inhibitors (Roche, Basel, Switzerland; Cat. #04906837001) and 0.1 µL of 1 mM of PMSF (phenylmethylsulfonyl fluoride). Each sample contained at least five retinas from three mice of the same group. Retinas were disintegrated using an aluminum shank and centrifuged for 30 min at 4 °C and 14,000 rpm. The supernatant was collected, and the protein concentration was determined using Quick Start™ Bovine Serum Albumin Standard 2 mg/mL (BIO-RAD, Hercules, CA, USA; Cat. #500-0206) and Quick Start™ Bradford 1× Dye Reagent (BIO-RAD, Hercules, CA, USA; Cat. #500-0205). A total of 20 µg was separated on 15% SDS-PAGE gel and transferred onto polyvinylidene difluoride (PVDF) membranes (BIO-RAD, Hercules, CA, USA; Cat. #1620177). Then, the membranes were blocked in Tris-buffered saline with Tween 20 (TBS-T) (20 mM of Tris-HCl pH 7.6, 150 mM of NaCl, and 0.1% Tween-20) containing 5% non-fat milk (BIO-RAD, Hercules, CA, USA; Cat. #170-6404) for 2 h at room temperature. Subsequently, the membranes were incubated in a blocking solution containing the same primary antibodies used for immunofluorescence assays (1:1000) and anti-GAPDH antibody (Cell Signaling, Danvers, MA, USA; Cat. #2118; 1:5000) overnight at 4 °C. Then, membranes were washed three times with TBS-T and were incubated with goat anti-rabbit peroxidase-conjugated secondary antibody (Cell Signaling, Danvers, MA, USA; Cat. #7074S; 1:7000) diluted in blocking solution (1:7000) for 2 h at room temperature. The proteins were detected by chemiluminescence using a SuperSignal™ West Femto Trial Kit (Thermo Fisher Scientific Inc., Wilmington, DE, USA; Cat. #34094) and radiographic plates (Carestream Dental; The Exchange, Atlanta, GA, USA; Cat. #MIN-REV). The plates were scanned, and the densitometry analysis was performed in ImageJ software (ImageJ2, National Institutes of Health, Bethesda, MD, USA).

### 4.6. Electroretinographic (ERG) Assessments

To assess the role of *Sgcd* in retinal function, we performed ERG assessments in both groups. Mice were initially maintained in a dark room overnight. On the experimentation day, we then anesthetized with 1 µL/g body weight intraperitoneally of a combination of 70% ketamine (1000 mg/10 mL) and 30% xylazine (2%). The pupils were dilated with 0.5% tropicamide and 0.5% phenylephrine. We recorded flash ERG responses from both eyes by a silver chloride ring electrode placed on the cornea. Two reference electrodes were positioned subcutaneously near the eyes. All procedures were performed under dim red light. The light stimulation included a 1-ms flash with an intensity of 0.3, 0.6, 0.9 and 1.2 log cd.s/m^2^ (PS33 Plus PhotoStimulator, GRASS Technologies, Warwick, RI, USA). The bandpass was set at 3 to 300 Hz (P511AC Amplifier, GRASS Technologies). We averaged 16 responses, as previously described in a work of ours [33]. We analyzed our data and calculated A and B-wave amplitudes and latency in MatLab (Mathworks, Natick, MA, USA) following a standardized approach. [34]

### 4.7. Statistical Analysis

To begin our approach, we verified the normal distribution of our data by plotting and Kolmogorov–Smirnov tests. Most of our data was normally distributed; hence, when the sample size was greater than 30 units of observation, we applied parametric methods to describe the effect of Sgcd or their lack of on several anatomic and electrophysiologic measures, e.g., means and standard deviations, Student’s *t*-test, ANOVA with Bonferroni-corrected *p*-values. However, when the sample size was <30 or the data was not normally distributed nor adequately transformed, we applied nonparametric statistics for all our inferences, e.g., medians and interquartile range, Wilcoxon two-sample test, and two-way Friedman’s ANOVA with Bonferroni-corrected pairwise comparisons.

## Figures and Tables

**Table 1:** Structural characteristics of three-month-old *Sgcd^+/+^* and *Sgcd^−/−^* mice. ^1^

Characteristic	*Sgcd^+/+^*	*Sgcd^–/–^*	*p*-Value ^1,2^
Retinal thickness (µm) ^1^	252.4 ± 51.5	149.0 ± 51.0	<0.0001
Differences any layer ^2^			<0.0001 ^a^
Photoreceptor	42.6 ± 15.6	24.7 ± 8.3	<0.0001 ^b^
Outer segment	109.6 ± 20.2	75.5 ± 15.0	<0.0001 ^b^
Inner segment	135.8 ± 22.4	83.1 ± 24.3	<0.0001 ^b^
Outer nuclear	67.0 ± 10.2	50.8 ± 11.8	<0.0001 ^b^
Outer plexiform	19.9 ± 5.3	11.2 ± 2.7	<0.0001 ^b^
Inner nuclear	49.4 ± 10.7	31.0 ± 10.1	<0.0001 ^b^
Inner plexiform	53.6 ± 12.7	32.9 ± 16.7	<0.0001 ^b^
Ganglion cell	12.9 ± 4.1	8.1 ± 4.1	n.s.
Nuclei density per 100 µm^2^			
Outer nuclear ^1^ Inner nuclear ^1^	2.5 ± 0.4 1.6 ± 0.3	2.7 ± 0.3 1.96 ± 0.4	0.0033 <0.0001
Solution of continuity in the H&E ^3^	13.0 ± 5.0	0.0 ± 3.0	<0.0001

^1^ Mean ± SD. Student’s *t*-test. ^2^ Mean ± SD. a: ANOVA and b: Student’s *t*-test pairwise comparisons with Bonferroni corrected *p*-values. ^3^ Median ± interquartile range (IQR). Wilcoxon two-sample test. n.s.: not significant at the 0.05 level.

**Table 2:** Differences in normalized protein expression of three-month-old *Sgcd^−/−^* and *Sgcd^+/+^* mice (*n* = 12). ^1^

Sg-Sspn Subunit	Median Difference (95% CI) *Sgcd^–/–^* vs. *Sgcd^+/+^*	*p*-Value	H_1_ Based on IF
α-sarcoglycan	0.1320 (0.0775, 0.2031)	0.0286	M_KO_ > M_WT_
β-sarcoglycan	−0.1598 (−0.1986, −0.0572)	0.0286	M_KO_ < M_WT_
γ-sarcoglycan	−0.7787 (−0.9732, −0.4996)	0.0286	M_KO_ < M_WT_
δ-sarcoglycan	−1.0802 (−1.5383, −0.8444)	0.0286	M_KO_ < M_WT_
ε-sarcoglycan	−0.0846 (−0.1108, −0.0260)	0.0286	M_KO_ < M_WT_
Sspn	−0.18043 (−0.3132, 0.0668)	0.1143	M_KO_ < M_WT_

^1^ Sg-Sspn: Sarcoglycan–sarcospan complex. IF: Indirect immunofluorescence staining. Median normalized protein expression among knock-out (M_KO_) and wild type (M_WT_).

## Supplementary Materials

Supplementary materials can be found at https://www.mdpi.com/1422-0067/20/21/5480/s1.

## Abbreviations

AMD	Age-related macular degeneration
ANOVA	Analysis of variance
ARVO	The Association for Research in Vision and Ophthalmology
DAPc	Dystrophin-associated protein complex
Dp71	Dystrophin isoform of 71 kilodaltons
ERG	Electroretinography or electroretinogram, as applicable
GCL	Ganglion cell layer
H&E	Hematoxylin and eosin
INL	Inner nuclear layer
IPL	Inner plexiform layer
KO	Knocked-out for *Sgcd^−/−^* gene
LGMD2	Limb-girdle muscular dystrophy
M ± IQR	Median ± interquartile range
ms	Millisecond
ONL	Outer nuclear layer
OPL	Outer plexiform layer
OR	Odds ratio
PBS	Phosphate-buffered saline
PR	Photoreceptor
RR	Risk ratio
Sg(s)	Sarcoglycan(s)
Sspn	Sarcospan(s)
Sgcd/SGCD	Delta-sarcoglycan murine protein (Lowercase) or human (Uppercase)
*Sgcd/SGCD*	Delta-sarcoglycan murine gene (Lowercase) or human (Uppercase)
µV	Microvolt
WT	Wild type for *Sgcd*^+/+^.
