# Supplementary material for: Lack of Delta-Sarcoglycan (Sgcd) Results in Retinal Degeneration
Source: Int J Mol Sci. 2019 Nov 4;20(21):5480. doi: 10.3390/ijms20215480 (PMC6862322; doi:10.3390/ijms20215480)
Supplement: Supplementary file 1 [file ijms-20-05480-s001.pdf]

SUPPLEMENTARY FIGURES

Figure S1. Alpha-sarcoglycan blot.

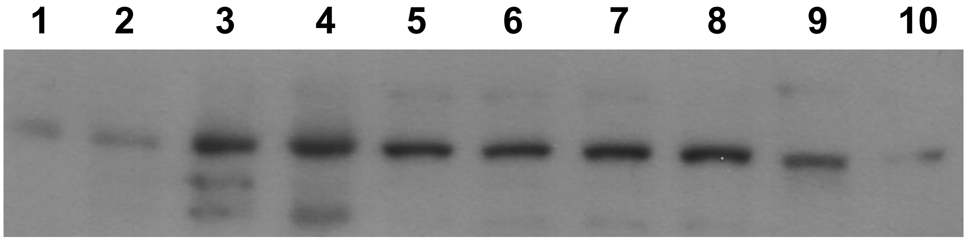

Lanes 1–4: Sgcd$^{+/+}$ dissected retinas.
Lanes 5–8: Sgcd$^{-/-}$ dissected retinas.
Lanes 9: Sgcd$^{+/+}$ muscle explants.
Lanes 10: Sgcd$^{-/-}$ muscle explants.

Figure S2. Beta-sarcoglycan blot.

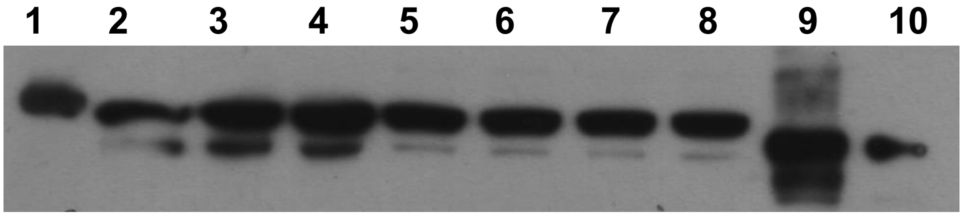

Lanes 1–4: Sgcd$^{+/+}$ dissected retinas.
Lanes 5–8: Sgcd$^{-/-}$ dissected retinas.
Lanes 9: Sgcd$^{+/+}$ muscle explants.
Lanes 10: Sgcd$^{-/-}$ muscle explants.

Figure S3. Gamma-sarcoglycan blot.

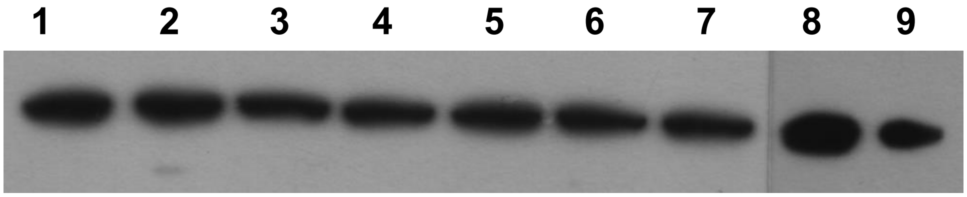

Lanes 1–3: Sgcd$^{+/+}$ dissected retinas.
Lanes 4–7: Sgcd$^{-/-}$ dissected retinas.
Lanes 8: Sgcd$^{+/+}$ muscle explants.
Lanes 9: Sgcd$^{-/-}$ muscle explants.

Figure S4. Delta-sarcoglycan blot.

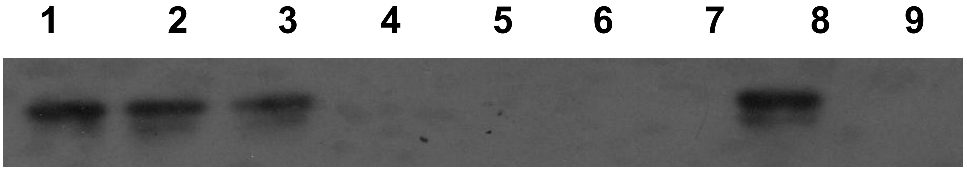

Lanes 1–3: Sgcd$^{+/+}$ dissected retinas.
Lanes 4–7: Sgcd$^{-/-}$ dissected retinas.
Lanes 8: Sgcd$^{+/+}$ muscle explants.
Lanes 9: Sgcd$^{-/-}$ muscle explants.

Figure S5. Epsilon-sarcoglycan blot.

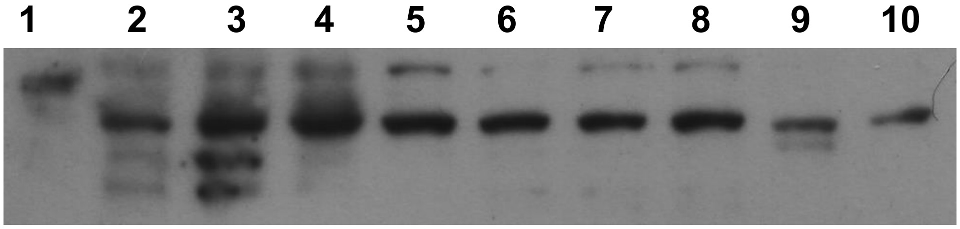

Lanes 1–4: Sgcd$^{+/+}$ dissected retinas.
Lanes 5–8: Sgcd$^{-/-}$ dissected retinas.
Lanes 9: Sgcd$^{+/+}$ muscle explants.
Lanes 10: Sgcd$^{-/-}$ muscle explants.

Figure S6. Sarcospan blot.

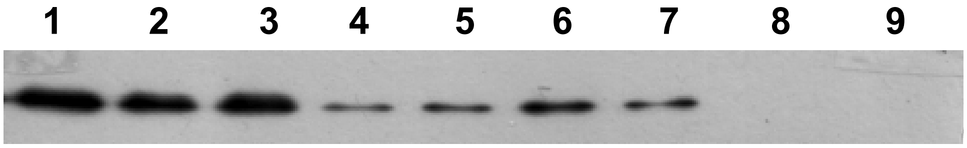

Lanes 1–3: Sgcd$^{+/+}$ dissected retinas.
Lanes 4–7: Sgcd$^{-/-}$ dissected retinas.
Lanes 8: Sgcd$^{+/+}$ muscle explants.
Lanes 9: Sgcd$^{-/-}$ muscle explants.

Figure S7. GADPH blot for protein normalization.

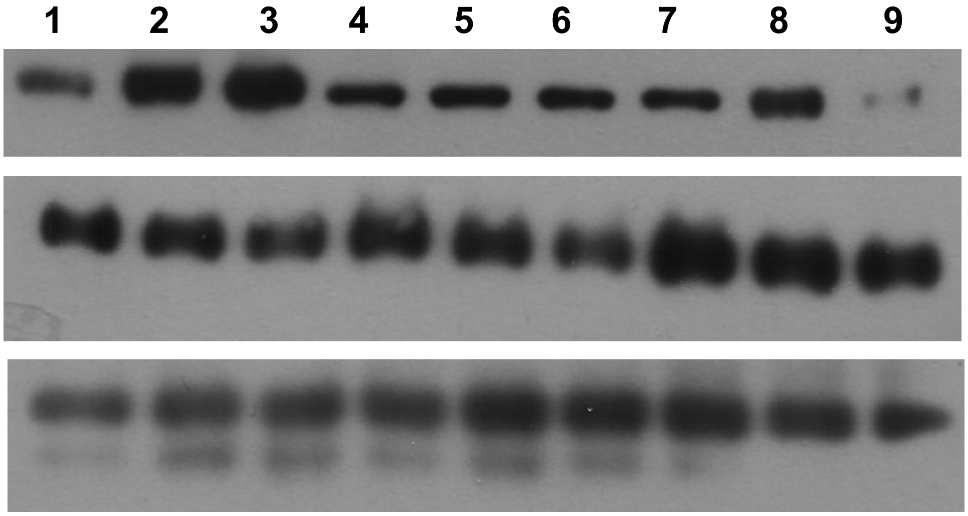

Lanes 1–3: Sgcd$^{+/+}$ dissected retinas.
Lanes 4–7: Sgcd$^{-/-}$ dissected retinas.
Lanes 8: Sgcd$^{+/+}$ muscle explants.
Lanes 9: Sgcd$^{-/-}$ muscle explants.
